# A Delirium Risk Stratification Tool and Interdisciplinary Rounds to Prevent Delirium in Hospitalized Older Adults

**DOI:** 10.1093/geroni/igab046.2279

**Published:** 2021-12-17

**Authors:** Jennifer Woodward, Tru Byrnes

**Affiliations:** Atrium Health, Charlotte, North Carolina, United States

## Abstract

Delirium is a disturbance of attention accompanied by a change in baseline cognition that is commonly seen in acute care settings, and effects up to 80% of ICU patients. The development of delirium has adverse effects on patient outcomes and high health care costs. Of patients aged 65+ admitted to our hospital in 2019, non-delirious patients had a five-day length of stay (LOS) compared to a 10-14 days LOS in delirious patients. A five days LOS increase adds an additional $ 8,325 per patient for an extra annual cost of 15 million dollars. Additionally, delirium is often not recognized. A prior retrospective study showed that 31% of older adults seen by a Geriatrics provider were diagnosed with delirium, while only 11% were detected by nurse’s CAM screen. Given the need to improve delirium detection and management, a QI project was undertaken with a goal to recruit an interdisciplinary team, create a risk stratification tool to identify patients at substantial risk for developing delirium, and develop a delirium prevention protocol. Patients with a score of ≥ 4 were initiated on a nurse driven delirium protocol that included a delirium precaution sign and caregiver education. 6 months data has shown increased delirium detection of 33%, a reduction in 7.7 days LOS, reduced SNF discharge by 27%, and a significant LOS saving of 231 days. The results were statistically significant, p < 0.04 for LOS reduction. The cost avoidance in LOS alone were $384,615 for delirium patients.

